# Targeted Molecular Therapy in Glioblastoma

**DOI:** 10.1155/2020/5104876

**Published:** 2020-01-14

**Authors:** Claudio Festuccia, Assunta Leda Biordi, Vincenzo Tombolini, Akira Hara, David Bailey

**Affiliations:** ^1^Department of Applied Clinical Sciences and Biotechnologies, University of L'Aquila, L'Aquila, Italy; ^2^Department of Experimental Medicine, University La Sapienza, Rome, Italy; ^3^Gifu University Graduate School of Medicine, Gifu, Japan; ^4^IOTA Pharmaceuticals Ltd., Cambridge University Biomedical Innovation Hub, Cambridge, UK

Glioblastoma multiforme (GBM) is the most common and malignant type of primary brain tumor, exhibiting poor response to therapy and seemingly inevitable recurrence. The current standard of care for treating newly diagnosed GBM is maximal safe resection followed by radiation with concurrent and adjuvant temozolomide, a regimen which has been shown to improve median overall survival (12.1 months vs. 14.6 months) and 2-year (27.2% vs. 10.9%) and 5-year (10.9% vs. 1.9%) survival rates when compared with radiation alone.

This special issue was aimed at updating researchers on current topics and progress made in basic, preclinical, and clinical glioblastoma research. It also provided a platform for pharmaceutical and translational scientists to submit original research articles, review articles, and clinical studies, focusing on the evaluation of new molecular pathways as pharmacological targets for treatment strategies which may improve the management of aggressive, drug-resistant GBM, in the hope that a deeper knowledge of GBM biology may eventually lead to effective targeted therapeutic approaches based on the inhibition of tumor-specific proteins or molecular pathways.

Unfortunately, this neoplasia contains an elevated percentage of transformed, self-maintaining, multipotent, tumour-initiating cancer stem cells, mainly present in highly hypoxic areas of the tumor in conjunction with palisading necrosis. Despite multimodal therapies, prognosis for GBM is still dismal. Many features contribute to this therapeutic challenge, including high intratumoral and intertumoral heterogeneity, resistance to therapy, migration and invasion, and immunosuppression. Nevertheless, with the advent of novel high-throughput drug screening technologies, together with a growing body of genetic and transcriptomic information, significant progress has been made to understand the molecular and immunological signatures underlying the pathology of glioblastoma.

We received 11 reports for publication, accepting 7 after peer review. A brief summary of all accepted papers is provided below.

Elevated invasive capacity is one of the key tumoral features associated with treatment resistance, recurrence, and poor overall survival in GBM. The research group of Akira Ara at the Gifu University Graduate School of Medicine (Gifu, Japan) reviewed treatment strategies based on histological targets against invasive and resistant GBM using the classification of the “secondary structures of Scherer.” One of the main reasons that gliomas are not cured by surgery is the topographically diffuse nature of the disease. In addition to the high degree of intratumor variability mentioned previously, the extensive spreading of malignant tumor cells within the brain parenchyma results in an inability to completely resect this tumor. Hans-Joachim Scherer was a pioneer in the study of glioma growth patterns. In 1940, Scherer described the appearance and behavior of glioma cells migrating away from the main tumor mass through the brain parenchyma. The patterns of glioma cell infiltration have since been referred to as the “secondary structures of Scherer.” Infiltrating glioma cells migrate through the normal parenchyma, collect just below the pial margin (subpial spread), surround neurons and vessels (perineuronal and perivascular satellitosis), and migrate through the white matter tracts (intrafascicular spread). Examples of observed secondary structures include perineuronal growth (perineuronal satellitosis), surface/subpial growth, perivascular growth, and intrafascicular growth. In order to develop therapeutic interventions to mitigate glioma cell migration, it is important to understand the biological mechanisms underlying the formation of these secondary structures. The review examined new molecular pathways based on the histopathological evidence of GBM invasion as a major prognostic factor in the high recurrence rate for GBMs. Specific molecular parameters, in addition to traditional histopathological analysis, have been used to define tumor classification in the revised 4th edition of the WHO Classification of CNS tumors, published in 2016. Detailed histopathological analysis based on the combination of molecular parameters with traditional analytical methods can now be used to evaluate efficacy of targeted therapies against cellular and genetic heterogeneity within both invasive and drug-resistant glioblastoma.

The molecular machinery underlying GBM invasiveness involves an intricate network of signaling pathways and interactions with the extracellular matrix and neighboring host cells. In this special issue, a collaboration amongst researchers from the Department of Neurological Surgery and Spine Unit and Genetics Unit (Hospital Universitario and Instituto de Investigación Marqués de Valdecilla in Santander, Spain), the Division of Neurosurgery (University of Toronto, Canada), and the MacFeeters-Hamilton Center for Neuro-Oncology Research (Princess Margaret Cancer Center in Toronto, Canada) is reported, reviewing and highlighting the molecular and clinical hallmarks of invasion in GBM. In this paper, C. Velásquez et al. review data on adhesion molecules, extracellular matrix (ECM) components, epithelial-to-mesenchymal transition (EMT), cytoskeleton-remodeling proteins, cross-talk with host cells and immune modulation, as well as the signaling pathways associated with GBM invasion (including those involving receptor tyrosine kinases, Wnt (both canonical and *β*-catenin-independent pathways), hedgehog-GLI1, and nuclear factor-*κ*B. The authors analyze the clinical implications of GBM invasiveness and assess GBM invasion in the clinical setting, by imaging GBM invasion intraoperatively to guide surgical resection and radiation therapy of the infiltrative tumor.

G. Steponaitis et al., Laboratory of Molecular Neuro-Oncology, Lithuanian University of Health Sciences (Kaunas, Lithuania), elucidated the oncosuppressive role of runt-related transcription factor 3 (RUNX3) in human astrocytomas. RUNX3 is a tumor suppressor gene whose inactivation was shown to be related to carcinogenesis in several cancers. Methylation status and protein expression levels of RUNX3 were measured by methylation-specific PCR and Western blotting in tissues harvested from normal tissues and glioma of different grades. These researchers demonstrate that RUNX3 gene methylation and protein expression downregulation are glioma malignancy-dependent and contribute to tumor progression. Importantly, these authors also demonstrated that re-expression of RUNX3 in the glioblastoma U87-MG cell line decreased cell viability *in vitro*. However, it remains to be demonstrated whether transfection of RUNX3 can reduce tumor progression and increase survival *in vivo* after injection of cells in nude mice.

E. Berney et al., Departments of Physiology/Anatomy and Pediatrics, University of North Texas Health Science Center (Fort Worth, Texas, USA), reported data on the scavenger receptor class B type 1 (SR-B1) as a potential target for treating glioblastoma. These studies involved the evaluation of reconstituted high-density lipoprotein (rHDL) nanoparticles (NPs) as delivery agents for the drug mammalian target of rapamycin (mTOR) inhibitor everolimus (EVR) to GBM cells. Cytotoxicity studies and assessment of downstream effects, including apoptosis, migration, and cell cycle events, were probed, in relation to the expression of SR-B1 by GBM cells. The authors revealed that rHDL/EVR formulation was 185 times more potent than free EVR against the high SR-B1 expressing GBM cell line LN 229. In addition, cell cycle analysis revealed that rHDL/EVR-treated LN229 cells had a 5.8 times higher apoptotic cell population than those treated with EVR. The sensitivity of GBM cells to EVR treatment was also strongly correlated with SR-B1 expression. So, delivering EVR and likely other agents, via a biocompatible transport system targeted to the SR-B1 receptor, could lead to effective personalized therapy of GBM.

C. Cilibrasi et al., School of Medicine and Surgery (University of Milano-Bicocca), in collaboration with the NeuroMI, Milan Center of Neuroscience, the Departments of Neurology and Neuroscience, San Gerardo Hospital, the Department of Neurology and Neurosurgery, Montreal Neurological Institute and Hospital (McGill University, Montreal, Quebec, Canada), the International Center for Digestive Health (ICDH), University of Milano-Bicocca, and the Genome Damage and Stability Center, School of Life Sciences, University of Sussex, UK, showed that a ploidy increase promotes sensitivity of glioma stem cells to Aurora kinase inhibition, investigating the effect of Aurora kinase inhibition in five glioma stem cell lines isolated from glioblastoma patients. As expected, cell lines responded to the loss of Aurora kinase with cytokinesis failure and mitotic exit without cell division. Surprisingly, this resulted in a proliferative arrest in only two of the five cell lines. Sensitive cell lines entered a senescent/autophagic state following aberrant mitotic exit, while the nonsensitive cell lines continued to proliferate. This senescence response did not correlate with TP53 mutation status but only occurred in the cell lines with the highest chromosome content. Repeated rounds of Aurora kinase inhibition caused a gradual increase in chromosome content in the resistant cell lines, eventually leading to a similar senescence response and proliferative arrest. The results suggest that a ploidy threshold is the main determinant of Aurora kinase sensitivity in TP53 mutant glioma stem cells. Thus, ploidy could be used as a biomarker for treating glioma patients with Aurora kinase inhibitors in TP53 mutant glioma stem cells. Further research will be necessary to explore the mechanism of ploidy-induced senescence and the precise reason why a particular ploidy threshold appears to trigger this response.

A. Menezes et al. investigated the impact of HDAC activity on GBM cell behavior and plasticity by live cell imaging. These researchers knocked down HDAC activity pharmacologically using two different inhibitors (TSA and SAHA) in two different tumor cell types: a commercial GBM cell line (U87-MG) and a primary tumor (GBM011). Upon 72 hours of *in vitro* HDAC inhibitor treatment, GBM cells presented a very unusual elongated cell shape due to the formation of tunneling tubes which appeared independent of TGF*β*-induced EMT. Live cell imaging revealed that voltage-sensitive Ca++ signaling was disrupted upon HDAC activity blockade. This behavior was coupled to downregulation of vimentin and connexin gene expression, suggesting that HDAC activity blockade downgrades GBM aggressiveness due to tumor cell competence and plasticity modulation *in vitro*. To investigate these effects, GBM oncospheres were xenografted into the chick developing neural tube. Remarkably, when placed within the developing neural tube, HDAC inhibitor-treated GBM cells ectopically expressed HNK-1, a tumor suppressor marker tightly correlated to increased survival of patients. These results describe, for the first time in the literature, the relevance of HDAC inhibition to *in vivo* tumor cell morphology and competence in an appropriate response to environmental cues. Ultimately, the results highlight the relevance of chromatin remodeling for tumor cell plasticity and shed light on the clinical targeting of the epigenome in GBM therapy.

The blood-brain barrier (BBB) is an anatomical functional unit created by characteristic endothelial cells forming blood vessels within the central nervous system. The main function of the BBB is protecting brain tissue from harmful elements present in the blood while still allowing the passage of substances necessary for metabolic functions. BBB endothelial cells form a continuous and nonfenestrated endothelium, sealed by occluding cellular junctions (tight junctions), whose compactness prevents the passage of hydrophilic and high-molecular weight substances from the blood to the brain parenchyma, performing filtration which is much more selective than that of endothelial cells in the capillaries of other parts of the body. Further structural features of the BBB include projections of astrocytic cells, called astrocytic peduncles (also known as the “glial limiting” membrane), which surround the endothelial cells of the BBB, providing an additional “barrier.” Although the structure of the BBB is often impaired in GBM, it is thought that BBB penetrance still represents a serious barrier to drug delivery to the tumor. Different methods have been exploited to bypass the BBB and increase the tumor uptake of therapeutic agents. In this issue, M. Shi and L. Sanche of the Department of Radiation Oncology, School of Medicine, Hangzhou, China and the Department of Nuclear Medicine and Radiobiology, Université de Sherbrooke, Canada, evaluated the efficacy of convection-enhanced delivery (CED), using multiple drugs with different antitumor mechanisms, concomitant with radiation and chemotherapy. Importantly, the simultaneous use of these procedures demonstrated supra-additive effects over standard drug treatments, representing a promising modality for brain tumor therapy. The authors also evaluated the efficacy of different CED-based strategies as part of Phase II and III clinical trials. CED bypasses the BBB, increases drug uptake by the tumor, and reduces systemic toxicity.

